# Burnout Subtypes and Absence of Self-Compassion in Primary Healthcare Professionals: A Cross-Sectional Study

**DOI:** 10.1371/journal.pone.0157499

**Published:** 2016-06-16

**Authors:** Jesus Montero-Marin, Fernando Zubiaga, Maria Cereceda, Marcelo Marcos Piva Demarzo, Patricia Trenc, Javier Garcia-Campayo

**Affiliations:** 1 Faculty of Health and Sport Sciences, University of Zaragoza, Zaragoza, Spain; 2 Primary Care Prevention and Health Promotion Research Network (RedIAPP), Zaragoza, Spain, and Centro de Investigación Biomédica en Red de Salud Mental, CIBERSAM, Spain; 3 Mente Aberta – Brazilian Center for Mindfulness and Health Promotion, Department of Preventive Medicine, Universidade Federal de Sao Paulo, UNIFESP, Sao Paulo, Brazil; 4 Miguel Servet Hospital and University of Zaragoza, Zaragoza, Spain; Cardiff University, UNITED KINGDOM

## Abstract

**Background:**

Primary healthcare professionals report high levels of distress and burnout. A new model of burnout has been developed to differentiate three clinical subtypes: ‘frenetic’, ‘underchallenged’ and ‘worn-out’. The aim of this study was to confirm the validity and reliability of the burnout subtype model in Spanish primary healthcare professionals, and to assess the explanatory power of the self-compassion construct as a possible protective factor.

**Method:**

The study employed a cross-sectional design. A sample of n = 440 Spanish primary healthcare professionals (214 general practitioners, 184 nurses, 42 medical residents) completed the Burnout Clinical Subtype Questionnaire (BCSQ-36), the Maslach Burnout Inventory General Survey (MBI-GS), the Self-Compassion Scale (SCS), the Utrecht Work Engagement Scale (UWES) and the Positive and Negative Affect Schedule (PANAS). The factor structure of the BCSQ-36 was estimated using confirmatory factor analysis (CFA) by the unweighted least squares method from polychoric correlations. Internal consistency (R) was assessed by squaring the correlation between the latent true variable and the observed variables. The relationships between the BCSQ-36 and the other constructs were analysed using Spearman’s r and multiple linear regression models.

**Results:**

The structure of the BCSQ-36 fit the data well, with adequate CFA indices for all the burnout subtypes. Reliability was adequate for all the scales and sub-scales (R≥0.75). Self-judgement was the self-compassion factor that explained the frenetic subtype (Beta = 0.36; p<0.001); isolation explained the underchallenged (Beta = 0.16; p = 0.010); and over-identification the worn-out (Beta = 0.25; p = 0.001). Other significant associations were observed between the different burnout subtypes and the dimensions of the MBI-GS, UWES and PANAS.

**Conclusions:**

The typological definition of burnout through the BCSQ-36 showed good structure and appropriate internal consistence in Spanish primary healthcare professionals. The negative self-compassion dimensions seem to play a relevant role in explaining the burnout profiles in this population, and they should be considered when designing specific treatments and interventions tailored to the specific vulnerability of each subtype.

## Background

Burnout syndrome is a profound consequence of chronic stress. It was originally observed in helping professions with personal interactions [1], although it has also come to be studied later in several types of occupations [2]. This syndrome is associated with a gradual loss of energy and enthusiasm [3], and has traditionally been defined by the dimensions of ‘exhaustion’, ‘cynicism’ and ‘inefficacy’ [4]. Exhaustion is the feeling of not being able to offer any more of oneself at work; cynicism represents a distant attitude towards work, those served by it and colleagues; inefficacy is the feeling of not performing tasks adequately. Burnout reflects a lack of harmony between the employee and his/her workplace [5], and develops progressively owing of the use of ineffective coping strategies with which workers attempt to protect themselves from the job-related stress [6]. It is a negative experience, comprising cognitions, emotions and behaviours towards work, towards the people who relate with the individual in his/her workplace, and towards his/her professional role [7]. It has been said that some internal states, such as guilt, cause a predisposition to suffering from burnout [8].

As an opposite to burnout, engagement has been described by the dimensions of ‘vigour’, ‘dedication’ and ‘absorption’, reflecting a positive and fulfilling work-related state of mind, in which workers show a great deal of energy, participation, and a sense of ability to cope with workplace requirements [9]. The job demand-resources model allows both burnout and engagement to be explained as a result of an interaction between job demands and individual resources, so that excessive demands might cause exhaustion [10], but adequate resources may mitigate the effect of demands, encouraging engagement [11]. However, there are limits to a healthy engagement; for instance, commitment may be excessive, leading to exhaustion and harming health [12, 13]. Some studies have indicated that the engagement dimension of absorption may be present in other disorders such as workaholism [14]. Therefore, engagement would drive a deep dive into the work [15], but under what circumstances engagement leads to positive or negative forms of performance still remains to be clarified [16].

Recently, a more comprehensive definition of burnout has been proposed to differentiate three clinical subtypes [17]. This typology emphasizes a phenomenological perspective and makes it possible to address the specific sources of subjective suffering [5, 18]. It groups distinct degrees of effort and persistence together in the same framework [17, 19], which is rooted in a differential pattern of coping styles [20, 21]. As described previously [22], the ‘frenetic’ subtype shows *ambition*, caused by their great need to achieve goals; *overload*, because they are risking their health and private life in the pursuit of good results; and *involvement*, in the sense that their efforts are increased to cope with difficulties in order to produce the expected results. With their active coping style focused on problems solving, the frenetic subtype represents a state of high motivation in which suffering is derived from their lack of ability to recognize their limitations [20, 23], and they feel guilty for not achieving set goals, given their great ambition [17, 22]. The ‘underchallenged’ subtype displays *indifference*, given that they perform their work-related tasks without interest and superficially; *lack of development*, as the absence of personal growth experiences together with their desire of take on other jobs; and *boredom*, given that they suffer from a lack of stimulus and monotony when performing tasks. With their escapist cognitive avoidance, the underchallenged subtype suffers from losing sight of the natural right of personal development [20, 23], and is invaded by feelings of guilt due to the ambivalence they feel for their work and by their desire for change [17, 22]. The ‘worn-out’ subtype presents *lack of acknowledgement* for the effort they have invested; *lack of control* over the results of their work; and *neglect*, as a preferential response to difficulties [17, 19]. With their passive coping style based on behavioural disengagement [20, 23], the worn-out subtype could trigger important health and motivation impairments, as well as feelings of guilt resulting from their not fulfilling adequately the responsibilities of their post [17, 22].

In recent years, there has been a growing movement that recognizes the potential role of compassion and self-compassion practices in many fields, such as healthcare. Western psychological tradition proposes that compassion is a complex construct that involves cognitive, affective and behavioural characteristics towards alleviating suffering from others and oneself [24]. In Eastern theories, compassion is a basic quality of human beings rooted in the recognition of and desire to alleviate suffering, and gives rise to pro-social behaviours [25]. Self-compassion is one of the two facets of compassion, in addition to compassion for others. It has been defined as being touched by and open to one’s own suffering, not avoiding or disconnecting from it, and generating the desire to alleviate one’s suffering and to heal oneself with kindness [26]. Suffering, in the contemporary application of the compassion construct, can be understood as daily life stressors such as the ageing process, illness and disease conditions, and also dysfunctional relationships and environments, including the workplace.

There are three theoretical facets to self-compassion, represented by pairs of opposing subscales: self-kindness vs self-judgement; common humanity vs isolation; and mindfulness vs over-identification. The self-kindness facet represents an alternative to self-blaming and self-criticism. Common humanity addresses equanimity that acknowledges human suffering is inherent to the nature of life and is associated with the suffering of others. Finally, the mindfulness facet represents curiosity and acceptance, rather than over-identification with difficulties and uncomfortable thoughts and experiences [27]. Self-compassion is associated with several aspects of positive mental health, and as a trainable quality, it seems to be useful as a protective factor for health professionals at risk of developing job-related stress [28–30].

Recent studies have found that 20–60% of physicians report burnout symptoms across all specialties, depending on the use of different measuring instruments, cut-off points and definitions [31–37], and that about one-third of primary care professionals may have high levels of this disabling syndrome [38]. In general, primary care personnel usually report high levels of distress related to job strain [39], which is an occupational hazard for healthcare providers, and has been related to poorer quality of care, worse patient safety and outcomes, low health status, greater intention to leave the practice, and high levels of burnout [40–43]. Burnout negatively affects the empathy, communication and reciprocity in the patient-professional relationship, and may produce certain propensity for medical errors and poorer outcomes for patients [44, 45]. Techniques for training self-compassion may improve patient trust, adequacy of prescriptions and care costs, which is important from a viewpoint of efficiency [46]. Self-compassion has also been related to some factors conceptually close to burnout and quality of care [47].

However, there are no studies assessing the burnout subtypes definition in primary care professionals, and there are no studies evaluating the explanatory power of the self-compassion construct regarding the burnout subtypes. Knowing whether the burnout subtypes model fits well to these workers, as well as observing possible relationships between self-compassion and the burnout subtypes, could offer clues to specific ways of improving the well-being and the quality of care provided by this group of professionals. This might be possible by reinforcing personal resources, promoting positive experiences, alleviating specific feelings of guilt and improving patient trust. The possible different connection between the self-compassion facets and each burnout subtype might provide useful information for developing programmes targeting each specific profile, efficiently administered through brief [48] or online [49] interventions. This kind of approach is relevant at an historical moment in which interventions at the organizational level in several health care systems (e.g., reducing workload) are greatly restricted by political and economic constraints as a result of the recent financial crisis [50]. In this context, the aim of this study was to evaluate the structure, consistence and convergence of the burnout subtypes model in a sample of Spanish primary healthcare professionals, and to assess the explanatory power of self-compassion on the different burnout subtypes.

We started out with the assumption that the greater the levels of effort and persistence at work (frenetic subtype), the more relationships there would be with the factors of engagement and positive affect, whereas the lower the levels of effort and persistence (worn-out subtype), the more relationships there would be with the traditional factors of burnout and negative affect. We also expected that the frenetic subtype would be the most related to the self-compassion components of lack of self-kindness and self-judgement, because of their own non-conformity as a way of functioning. The underchallenged subtype would be the most associated with lack of common humanity and isolation, because of their tendency towards a lack of personal growth in a job in which recognizing others must be inherent. The worn-out subtype would be the most linked to lack of mindfulness and of over-identification, owing to their apparent lack of acceptance of negative thoughts and experiences.

## Methods

### Design

The correlation method was used, with a cross-sectional design based on individual differences for data collection. Measurements were obtained by a self-reported questionnaire on paper.

### Participants, procedure and ethics

The study population consisted of Spanish primary healthcare professionals (physicians, nurses and medical residents), who were employed from May to July 2015 in the district of Zaragoza, Spain (N = 1,579). The sample size was calculated for a 95% confidence interval, with a 4.0% error, and assuming a prevalence of burnout to be around 33% of primary care personnel, according to previous studies on the population of Spanish primary healthcare professionals [38]. The calculation yielded a result of 400 subjects. This sample size was in line with the recommended 10:1 ratio for the number of subjects to the number of test items, as a construct validity evaluation criterion [51], so that it was psychometrically adequate for the study. As we expected a loss of 10% from missing data, we inflated the numbers to reach a total sample size of around 440 participants. They were chosen by means of random stratified sampling with proportional allocation, depending on occupation (physicians: 48.5%; nurses: 41.9%; residents: 9.6%), from an alphabetical list of the total workforce. In case of refusal to participate, the following worker on the list was contacted, with the process continuing until the referred sample size was achieved. The questionnaire and protocols were approved by the Ethics Committee for Clinical Research of Aragon, Spain, and participants gave their informed consent attesting to their willingness to participate.

### Measures

#### Socio-demographic and occupational characteristics

Background information from the participants included: age, sex, stable relationships (yes or not), children (none vs one or more), occupation (physician, nurse, resident), worked hours per week, length of service, time at the same workplace, contract duration (temporary vs permanent), contract type (full-time vs part-time), economic difficulties (never, sometimes, almost always, always), sick leave (yes vs no), and days of sick leave in the previous year.

#### Burnout Clinical Subtypes Questionnaire (BCSQ-36)

The BCSQ-36 [19] consist of 36 items distributed into 3 scales and 9 subscales (S1 Fig). The ‘frenetic’ scale assesses ‘involvement’ (e.g. ‘I react to difficulties in my work with greater participation’), ‘ambition’ (e.g. ‘I have a strong need for important achievements in my work’), and ‘overload’ (e.g. ‘I overlook my own needs to fulfil work demands’). The ‘underchallenged’ scale consists of ‘indifference’ (e.g. ‘I feel indifferent about my work and have little desire to succeed’), ‘lack of development’ (e.g. ‘My work doesn’t offer me opportunities to develop my abilities’), and ‘boredom’ (e.g. ‘I feel bored at work’). The ‘worn-out’ scale comprises ‘neglect’ (e.g. ‘When things at work don’t turn out as well as they should, I stop trying’), ‘lack of acknowledgement’ (e.g. ‘I think my dedication to my work is not acknowledged’), and ‘lack of control’ (e.g. ‘I feel the results of my work are beyond my control’). Subjects had to rate their degree of agreement with each one of the statements according to a Likert 7-point scale, scoring between 1 (‘totally disagree’) and 7 (‘totally agree’). The ratings are presented as scalar scores, with adequate structure and reliability in other populations such us university workers [19].

#### Maslach Burnout Inventory General Survey (MBI-GS)

Subjects were asked to complete the MBI-GS [52], in its validated Spanish version [53]. This adaptation consists of 15 items grouped into the dimensions of: ‘exhaustion’ (e.g. ‘I feel emotionally drained from my work’; with α = 0.87 in our study); ‘cynicism’ (e.g. ‘I’ve become more callous toward people since I took this job’; α = 0.78); ‘efficacy’ (e.g. ‘I deal very effectively with the problems of my work’; α = 0.75). Responses were arranged on a Likert-type scale with seven response options, scored between 0 (‘never’) and 6 (‘always’).

#### Utrecht Work Engagement Scale (UWES)

The UWES [9] assesses a mental state of accomplishment, as an antithesis to burnout. This questionnaire consists of 17 items grouped into the factors of: ‘vigour’ (e.g. ‘At my work, I feel bursting with energy’; with α = 0.82 in our study), ‘dedication’ (e.g. ‘I find the work that I do full of meaning and purpose’; α = 0.83), and ‘absorption’ (e.g. ‘Time flies when I am working’; α = 0.76). The validated Spanish version of the instrument was used, in which responses were arranged on a Likert-type scale with seven response options, scored between 0 (‘never’) and 6 (‘always’).

#### The Positive and Negative Affect Schedule (PANAS)

The PANAS [54] is a self-report instrument that measure positive and negative affect. This questionnaire consists of a list of 20 adjectives, 10 per subscale (e.g. positive: ‘interested’, with α = 0.90 in our study; e.g. negative: ‘guilty’, α = 0.91), rated on a 5-point scale, and using the time instructions desired by the researcher. Present moment instructions were used in this study. This questionnaire has been validated in Spanish with good psychometric properties [55].

#### Self-Compassion Scale (SCS)

The SCS [27] is a 26-item questionnaire designed to assess self-compassion across: ‘self-kindness’ (e.g. ‘I try to love myself when I’m feeling emotional pain’; with α = 0.84 in our study), ‘self-judgement’ (e.g. “I’m disapproving and judgemental of my flaws and inadequacies’; α = 0.76), ‘common humanity’ (e.g. ‘I try to see my failures as part of the human condition”; α = 0.72), ‘isolation’ (e.g. ‘When I’m feeling down, I tend to feel like most other people are happier than I am’; α = 0.71), ‘mindfulness’ (e.g. ‘When something upsets me, I try to keep my emotions in balance’; α = 0.80), ‘over-identification’ (e.g. ‘When I’m feeling down, I tend to obsess and fixate on everything that is going wrong’; α = 0.70). The items assess how respondents perceive their actions toward themselves in difficult times and are rated using a Likert-type scale anchored between 1 (‘almost never’) and 5 (‘almost always’). The Spanish [56] version of the SCS was used.

### Statistical analysis

Means (standard deviations), medians (interquartile ranges) and frequencies (percentages), were calculated for the socio-demographics. Moreover, skewness, kurtosis, and Mardia’s coefficients [57] were estimated to evaluate the items’ distribution. We used polychoric matrices to estimate a truer correlation between the theorized continuous latent variables from the observed ordinal items, and we estimated the characteristics of the matrices by the determinant, KMO index, and Barlett’s test of sphericity [58]. The determinant shows to what extent the variables are related, and it is zero when they are linearly dependent. The KMO index of sampling adequacy predicts whether data are likely to factor well (KMO should be ≥0.70 to proceed with factor analysis). Barlett’s test of sphericity checks whether the data comes from the identity matrix, with ones on the main diagonal and zero covariances elsewhere, or whether there is a redundancy between the variables that we can summarize with a lower number of factors.

As conducted in a previous study [59], we examined the fit of the models by confirmatory factor analysis (CFA), applying the Unweighted Least Squares method (ULS) [60]. The ULS does not provide inferential estimations based on the χ^2^ distribution, and therefore does not provide p-values. However, it does not require any distributional assumptions; it is robust and usually converges because of its efficiency in terms of computation; it tends to provide less biased estimates of the true parameter values than other procedures; and it shows good performance when working with polychoric matrices [61–64]. We used the goodness-of-fit index (GFI), the adjusted goodness-of-fit index (AGFI), the standardized root mean square residuals (RMSR), the normed fit index (NFI), and Bollen’s relative fit index (RFI). GFI and AGFI refer to explained variance, and values >0.90 are acceptable [65]. RMSR is the standardized difference between the observed and the predicted covariance, indicating good fit values <0.08 [66]. NFI measures the proportional reduction in the adjustment function when going from null to the proposed model, and is considered acceptable when >0.90 [67]. RFI takes into account the discrepancy for the model evaluated and for the baseline model, and is very good close to 1 [68]. First- and second-order standardized factor saturations (λ and γ), were also considered.

We examined the internal consistency of the scales using congeneric, tau-equivalent and parallel models of reliability [69]. The congeneric model assumes that each item measures the same latent variable, with possibly different scales, degrees of precision and magnitude of error. The tau-equivalent model implies that items measure the same latent variable, on the same scale, and with the same degree of precision, but with possibly different degrees of error. The parallel model assumes that all items must measure the same latent variable, on the same scale, with the same degree of precision, and with the same amount of error. In order to reach parsimony, we chose the most restrictive model that fit best with the data when applying the ULS procedure [70]. The reliability value was calculated by squaring the implied correlation between the composite latent true variable and the composite observed variable, to arrive at the percentage of the total observed variance that was accounted for by the true variable [70]. Item-rest and mean item-rest correlations were also calculated.

We used BCSQ scores in order to evaluate the degree of association regarding the other constructs, by means of Spearman’s r coefficients. Standardized coefficients (beta) were used to assess the individual contribution of the self-compassion factors to explain the different burnout subtypes, and the Wald test was used to evaluate their significance. Adjusted multiple determination coefficients (R^2^_y.123_) were also calculated to observe their grouped explanatory power, and their significance were assessed by means of analysis of variance [71]. Partial correlation coefficients (R_y3.12_) were estimated, indicating the correlation between two variables when the effect of the other variables is removed. Semi-partial correlation coefficients (R_y(3.12)_) were also calculated, the square of which shows the increase in the coefficient of determination after including a specific variable. The K-S test was used to determine whether the conditional distribution of the residuals met the assumption of normality. Finally, it was confirmed that the Durbin-Watson values (DW) approached a value ≈2.00 to rule out autocorrelation problems in the errors [71]. All of the tests used were bilateral and the significance level was α <0.05 SPSS v19, FACTOR v10 and AMOS v20 packages were used to conduct the statistical analysis.

## Results

All materials used to produce these results are available upon request, including a detailed list of documents and data files needed in order for replication, and what steps and in what sequence the interested researchers had to take in order to make this data available [72]. In addition, authors will post these materials in the group’s website [73].

### Characteristics of participants

There were 440 participants (response rate = 50.1%); the majority were women (70.3%) in their early fifties (mean = 49.67; SD = 10.86), who were in a relationship (76.6%) and with a child (median = 1; Q_1_-Q_2_ = 0–2). 214 subjects were physicians; 184 were nurses; and 42 were residents. The mean lifetime length of permanence in the primary care service was 24.65 years (SD = 11.75), with 10.38 years (SD = 10.24) spent at the last workplace. Around two-thirds (68.8%) had permanent contract and almost all (96.2%) worked full-time. They worked roughly 40 hours/week (mean = 40.99; SD = 8.16), and two-thirds (67.7%) never had economic difficulties. 17.7% took sick leave the previous year, with a mean of 9.04 (SD = 31.36) days.

### Factorial structure

The matrices showed adequate features [frenetic (Mardia’s = 27.73; p<0.001; KMO = 0.85; Bartlett χ^2^ = 1,956.30; df = 66; p<0.001; determinant = 0.008); underchallenged (Mardia’s = 66.41; p<0.001; KMO = 0.94; Bartlett χ^2^ = 2,863.30, df = 66, p<0.001; determinant<0.001); worn-out (Mardia’s = 39.63; p<0.001; KMO = 0.88; Bartlett χ^2^ = 2,119.10; df = 66; p<0.001; determinant = 0.005). Table 1 shows the psychometric characteristics of the items, factors and scales of the BCSQ-36. The standardized weights of the items and first-order factors were >0.5. All the solutions explained a large amount of the variance (frenetic: 64.0%; underchallenged: 73.0%; worn-out: 66.1%), and presented a very good fit to the data (S2 Fig).

**Table 1:** Psychometric characteristics of the BCSQ-36.

Scale/Factor/Item	Mn	SD	Skew	Kurt	λ (γ)	Item-rest (factor)	Item-rest (scale)
**Frenetic**	3.89	0.84	0.05	1.15			
Ambition	3.62	1.15	0.17	0.16	(0.93)		
bcsq 1	3.51	1.40	0.01	-0.53	0.68	0.70	0.53
bcsq 4	3.92	1.41	-0.10	-0.50	0.71	0.67	0.58
bcsq 7	3.70	1.33	0.08	-0.27	0.79	0.71	0.65
bcsq 10	3.33	1.35	0.31	-0.08	0.85	0.74	0.68
Overload	3.11	1.17	0.50	0.40	(0.51)		
bcsq 2	3.72	1.54	0.18	-0.50	0.64	0,57	0.45
bcsq 5	2.63	1.48	0.92	0.59	0.78	0.63	0.52
bcsq 8	2.92	1.49	0.65	0.09	0.79	0.68	0.53
bcsq 11	3.18	1.41	0.46	-0.11	0.65	0.57	0.45
Involvement	4.93	0.92	-0.64	1.79	(0.64)		
bcsq 3	5.47	1.09	-0.94	1.97	0.54	0.49	0.40
bcsq 6	4.79	1.33	-0.39	-0.22	0.69	0.56	0.52
bcsq 9	4.78	1.25	-0.88	1.14	0.71	0.59	0.49
bcsq 12	4.67	1.16	-0.64	0.99	0.67	0.57	0.46
**Underchallenged**	2.45	0.97	0.53	0.17			
Indifference	2.15	0.94	0.70	0.27	(0.89)		
bcsq 13	2.25	1.18	1.16	2.07	0.66	0.54	0.52
bcsq 16	2.19	1.22	1.33	2.36	0.84	0.71	0.55
bcsq 19	2.24	1.21	0.85	0.43	0.85	0.65	0.68
bcsq 22	1.94	1.06	1.48	3.82	0.79	0.63	0.69
L. Development	2.54	1.15	0.61	0.09	(0.94)		
bcsq 14	2.40	1.44	0.98	0.59	0.67	0,60	0.77
bcsq 17	2.38	1.21	0.94	1.36	0.84	0.70	0.70
bcsq 20	2.66	1.49	0.69	-0.31	0.85	0.77	0.71
bcsq 23	2.74	1.47	0.71	-0.15	0.74	0.61	0.77
Boredom	2.63	1.13	0.65	0.62	(0.98)		
bcsq 15	2.83	1.49	0.62	-0.12	0.74	0.68	0.78
bcsq 18	2.80	1.41	0.71	0.19	0.77	0.71	0.62
bcsq 21	2.58	1.28	0.72	0.21	0.84	0.75	0.66
bcsq 24	2.33	1.21	0.84	0.77	0.80	0.68	0.74
**Worn-out**	3.34	0.92	-0.11	-0.07			
L. Acknowledgement	3.88	1.26	0.42	1.57	(0.80)		
bcsq 25	2.88	1.42	0.68	0.18	0.54	0.39	0.47
bcsq 28	4.13	1.77	-0.04	-0.94	0.73	0.60	0.53
bcsq 31	4.47	1.61	-0.26	-0.67	0.68	0.60	0.66
bcsq 34	3.94	1.54	0.10	-0.62	0.76	0.66	0.60
Neglect	2.37	0.89	0.48	1.06	(0.55)		
bcsq 26	2.43	1.17	1.08	2.04	0.85	0,65	0.49
bcsq 29	2.41	1.09	0.76	1.35	0.78	0.69	0.62
bcsq 32	2.15	0.99	0.88	2.32	0.72	0.70	0.54
bcsq 35	2.49	1.09	0.61	0.82	0.74	0.62	0.44
L. Control	3.88	1.24	-0.05	0.50	(0.99)		
bcsq 27	3.80	1.56	0.10	-0.63	0.75	0.66	0.66
bcsq 30	4.14	1.70	-0.14	-0.89	0.73	0.62	0.61
bcsq 33	3.29	1.47	0.29	-0.57	0.74	0.62	0.48
bcsq 36	3.87	1.50	-0.06	-0.64	0.64	0.62	0.55

Mn: Mean. SD: Standard Deviation. Skew: skewness. Kurt: kurtosis Item-rest: item rest coefficient. λ: factorial weight.

### Internal Consistence

The frenetic and worn-out scales fitted with the congeneric model of reliability, while the underchallenged scale fitted with the parallel model (R = 0.84, R = 0.88 and R = 0.94, respectively) (S2 Fig). The lack of acknowledgement factor showed a congeneric model, while the rest of factors fitted with the parallel model (R≥0.75 in all of cases). The average of item-rest (scale) values for the frenetic subtype was 0.52, with 0.68 for the underchallenged subtype, and 0.55 for the worn-out subtype. The mean of item-rest (factor) for ambition was 0.71, with 0.61 for overload, 0.55 for involvement, 0.63 for indifference, 0.67 for lack of development, 0.71 for boredom, 0.56 for lack of acknowledgement, 0.67 for neglect, and 0.63 for lack of control.

### Convergent/divergent validity

The frenetic subtype presented the fewest (direct in all cases) relationships with the standard burnout definition [exhaustion (r = 0.28; p<0.001); cynicism (r = 0.12; p<0.049); efficacy (r = 0.18; p<0.001)]. The underchallenged subtype was placed in the middle [exhaustion (r = 0.38; p<0.001); cynicism (r = 0.45; p<0.001); efficacy (r = -0.25; p<0.001)]. The worn-out subtype was the more convergent in classical terms [exhaustion (r = 0.52; p<0.001); cynicism (r = 0.48; p<0.001); efficacy (r = -0.32; p<0.001)]. The frenetic was directly associated with engagement, positive affect and negative affect (p<0.001), while the underchallenged and worn-out were inversely related to engagement and positive affect, and directly related to negative affect (p<0.001). The relationships of the BCSQ-36 with the other constructs are shown in Table 2.

**Table 2:** Convergent/divergent validity of the BCSQ-36.

*Scale / factor*	Exhaustion	Cynicism	Efficacy	Vigor	Dedication	Absorption	Positive	Negative
**Frenetic**	0.28***	0.12*	0.18***	0.28***	0.28***	0.30***	0.24***	0.24***
Ambition	0.06	-0.01	0.18***	0.22***	0.29***	0.21***	0.28***	0.12*
Overload	0.45***	0.24***	-0.07	0.06	0.05	0.20***	0.07	0.34***
Involvement	0.08	-0.02	0.35***	0.38***	0.34***	0.32***	0.21***	0.03
**Underchallenged**	0.38***	0.45***	-0.25***	-0.34***	-0.46***	-0.30***	-0.18***	0.25***
Indifference	0.30***	0.38***	-0.26***	-0.34***	-0.39***	-0.27***	-0.18***	0.21***
L. Development	0.32***	0.39***	-0.17***	-0.28***	-0.39***	-0.24***	-0.12*	-0.22***
Boredom	0.36***	0.44***	-0.25***	-0.31***	-0.46***	-0.29***	-0.19***	-0.21***
**Worn-out**	0.52***	0.48***	-0.32***	-0.31***	-0.36***	-0.20***	-0.29***	0.29***
L. Acknowledgement	0.38***	0.33***	-0.13**	-0.14**	-0.21**	-0.09	-0.17***	0.19***
Neglect	0.32***	0.35***	-0.35***	-0.39***	-0.39***	-0.28***	-0.32***	0.22***
L. Control	0.51***	0.45***	-0.23***	-0.27***	-0.31***	-0.15**	-0.24***	0.29***

Values are Pearson’s r.

*** p<0.001;

** p<0.01;

* p<0.05

### Explanatory power of self-compassion

The explanatory power of the self-compassion factors on the burnout subtypes was significant (Table 3). The most explained burnout subtype was the frenetic (R^2^_y.123_ = 0.18; p<0.001), whilst the least explained was the underchallenged (R^2^_y.123_ = 0.05; p = 0.006), with the worn-out in the middle (R^2^_y.123_ = 0.11; p<0.001). The main self-compassion factor that contributed to explaining the frenetic was self-judgement (Beta = 0.36; p<0.001). Isolation was the main self-compassion factor explaining the underchallenged (Beta = 0.16; p = 0.010). The worn-out subtype was mainly explained by over-identification (Beta = 0.25; p = 0.001). Standard errors, residual distributions and DW values were appropriate, making it possible to go ahead with the regression.

**Table 3:** Explanatory power of self-compassion with regard to the burnout types.

**Frenetic**		R_y.123_	R^2^_y.123_	F (df_1_ / df_2_) p^a^	Se	DW	p^b^
		0.41	0.18	13.29 (6 / 399) <0.001	0.75	1.92	0.250
	R	R_y3.12_	R_y(3.12)_	B (95% CI)	Se	Beta	p^c^
*Intercept*				*1*.*89 (1*.*27–2*.*51)*	*0*.*31*		*<0*.*001*
Self-kindness	-0.06	0.02	0.01	0.01 (-0.03–0.04)	0.02	0.02	0.816
Self-judgement	0.39	0.26	0.25	0.09 (0.06–0.12)	0.02	0.36	<0.001
Humanity	0.13	0.08	0.07	0.02 (-0.01–0.05)	0.01	0.08	0.134
Isolation	0.21	0.02	0.02	0.01 (-0.03–0.05)	0.02	0.03	0.653
Mindfulness	0.02	0.04	0.03	0.01 (-0.02–0.05)	0.02	0.05	0.475
Overidentification	0.28	0.03	0.02	0.01 (-0.03–0.05)	0.02	0.04	0.594
**Underchallenged**		R_y.123_	R^2^_y.123_	F (df_1_ / df_2_) p^a^	Se	DW	p^b^
		0.22	0.05	3.06 (6 / 408) 0.006	0.95	1.65	0.189
	R	R_y3.12_	R_y(3.12)_	B (95% CI)	Se	Beta	p^c^
*Intercept*				*2*.*19 (1*.*41–2*.*97)*	*0*.*40*		*<0*.*001*
Self-kindness	-0.11	-0.10	-0.09	-0.04 (-0.08–0.01)	0.02	-0.14	0.056
Self-judgement	0.09	-0.05	-0.05	-0.02 (-0.06–0.02)	0.02	-0.07	0.299
Humanity	-0.03	-0.01	-0.01	<0.01 (-0.04–0.03)	0.02	-0.01	0.858
Isolation	0.15	0.13	0.12	0.07 (0.02–0.12)	0.03	0.16	0.010
Mindfulness	-0. 09	0.03	0.03	0.01 (-0.03–0.06)	0.02	0.04	0.602
Overidentification	0.15	0.05	0.05	0.03 (-0.03–0.08)	0.03	0.08	0.304
**Worn-out**		R_y.123_	R^2^_y.123_	F (df_1_ / df_2_) p^a^	Se	DW	p^b^
		0.33	0.11	7.40 (6 / 405) <0.001	0.87	1.85	0.853
	R	R_y3.12_	R_y(3.12)_	B (95% CI)	Se	Beta	p^c^
*Intercept*				*2*.*67 (1*.*95–3*.*39)*	*0*.*37*		*<0*.*001*
Self-kindness	-0.15	-0.07	-0.07	-0.03 (-0.06–0.01)	0.02	-0.11	0.168
Self-judgement	0.18	-0.04	-0.04	-0.02 (-0.05–0.02)	0.02	-0.06	0.397
Humanity	0.04	0.06	0.06	0.02 (-0.01–0.05)	0.02	0.07	0.196
Isolation	0.18	0.05	0.05	0.02 (-0.02–0.07)	0.03	0.06	0.322
Mindfulness	-0.15	-0.02	-0.02	-0.01 (-0.05–0.03)	0.02	-0.03	0.634
Overidentification	0.30	0.17	0.16	0.08 (0.04–0.13)	0.02	0.25	0.001

R = Pearson’s correlation. R_y.123_ = multiple correlation coefficient. R^2^_y.123_ = coefficient of multiple determination. adj-R^2^_y.123_ = adjusted coefficient of multiple determination. p^a^ = p value for variance analysis associated with the regression. Se = standard error. DW = Dubin-Watson value. p^b^ = p value for K-S test for normality contrast on residuals. R_y3.12_ = partial correlation coefficient. R_y(3.12)_ = semi-partial correlation coefficient. T = tolerance value. B = regression slope. CI = confidence interval. Beta = standardized slope. p^c^ = p value of Wald test result.

## Discussion

The present study is the first of its kind to assess the burnout clinical subtype definition in primary healthcare professionals and to explore potential associations with the self-compassion construct. This is relevant, because this particular population of health workers is highly vulnerable to burnout syndrome, and the association of the syndrome with different self-compassion facets may be a guide for future lines of treatment, adjusted to the vulnerability of the different burnout profiles. The main limitation of this study was the cross-sectional design, which did not allow us to establish causal relationships between the variables, although it served as a heuristic procedure to facilitate the generation of future hypotheses for intervention designs. We also have to keep in mind that the procedure used with self-report questionnaires, mediated by the levels of awareness and the willingness to participate, could bias the results either by exaggerating the reporting of symptoms in some participants or by reducing the presence of precisely those most affected workers. On the other hand, the main strength was the large sample size used, enabling an adequate use of the analytical models in psychometric terms. The sample was recruited in primary care settings, with professionals in different job positions and a high risk of burnout, therefore improving the relevance of the results.

The multivariate distribution of the BCSQ-36 items recommended calculating polychoric correlations for the CFA. In general, the behaviour of the items was adequate, with high and positive item-rest values for both factors and scales. All the items weighted strongly and positively in their corresponding factors, as well as the factors did in their corresponding scale, so that all the burnout subtype models fit the data well, explaining a great deal of the variance. Each item measured the corresponding factor with the same degree of precision and amount of error, except for lack of acknowledgement, something that may be explained because this factor refers to different sources of gratification, those from the organization and from receptors of the service [17, 19]. However, the internal consistence values were appropriate in all of factors, and they were also in the case of the scales. Except for the underchallenged subtype, which fit the parallel model, the others subtypes seemed to be being measured with possibly different scales, degrees of precision and levels of error. This result could mean that the definition of burnout raised by the underchallenged subtype seems to be less extensive than the other burnout subtype definitions. In other words, the factors of the underchallenged subtype might be closer than those belonging to the frenetic and worn-out subtypes. It has been said that the main characteristics of the subtypes may be overload in the case of the frenetic, lack of personal development in the case of the underchallenged, and neglect, in the case of the worn-out [74, 75]. Future research is needed in order to clarify the extensiveness of each burnout subtype definition, using samples of other health-related professionals or caregivers.

With regard to the scalar scores obtained for the burnout profiles, we have seen that the lowest value was that corresponding to the underchallenged subtype, and the highest value was for the frenetic subtype. Nevertheless, as we have observed according to the models of reliability, the different subtypes may be being measured in different scales, so that the comparison of them could be problematic. To overcome this limitation we compared the same subtype with different populations, such as university workers [19], observing that all of the scores were relatively low and under the levels shown by this other Spanish population. More differences between them were observed in the underchallenged profile. Therefore, primary healthcare professionals seem to experience quite good personal development in a job where interpersonal empathy, communication and reciprocity are keystones in the patient-professional relationship and quality of care [44, 45]. On the contrary, fewer differences were found in the frenetic subtype, so primary healthcare professionals could possibly be showing more the consequences related to the pressure under which they work, with overloaded agendas and services [76].

We found that all the burnout subtypes, in terms of their general scales, were significantly related to all the considered constructs, although we have seen that they showed different patterns of relationships. In line with our initial hypothesis, the frenetic subtype was directly related to the engagement dimensions, while the underchallenged and the worn-out were inversely related. In the same way, the frenetic subtype was less associated with burnout, while the underchallenged and worn-out, respectively, were more related to the syndrome. This apparent contradiction, which places the frenetic conglomerate in an awkward place, points it out as a possible explanation for other findings that indicate a sort of overlapping with other constructs, such as workaholism [77]. The frenetic subtype, at least in their behavioural component of working hard, might be showing the compulsiveness and extrinsic motivation of a non-adaptive state of commitment [15], bringing to light the complex and not entirely positive meaning of engagement, and the need for further research at this point [78, 79]. Interestingly, the underchallenged subtype was the one with the highest inverse associations with engagement, while the worn-out subtype was the one with the highest direct associations with burnout. This result suggests that burnout and engagement may not be exactly opposite poles of the same construct, but they might represent different concepts, with distinct implications [80]. Finally, the underchallenged and the worn-out subtypes were directly related to negative affect, and indirectly to positive affect, as expected, with higher values for the worn-out because of their theoretical lower levels of effort and persistence [17, 19]. On the contrary, the frenetic profile was directly related to both positive and negative affect, indicating an uncomfortable state of commitment as an intersection between the previously mentioned constructs.

The ingredients of self-compassion were differently connected to each burnout profile, and they were as hypothesized, although we found significant relationships only with the negative facets of the construct. The frenetic subtype was the best explained, perhaps for being the more present in the sample, and it was related to self-judgement. The underchallenged subtype was the worst explained, having the lowest presence, and it was associated with isolation. The worn-out subtype was related to over-identification. First of all, these results suggests that only the negative facets of self-compassion might be really important as true marks of vulnerability on the burnout subtypes, as has recently been described for psychopathological diseases in general [81]. Secondly, this finding adds rich information to open the possibility of introducing new specific components for the treatment of each burnout subtype. In this sense, and lastly, the frenetic profile seems to be suffering from their exaggerated non-conformity, and this might be related to the lack of recognition of their own limitations, maybe because of a rigid performance-based self-esteem, which could facilitate the emergence of exhaustion and feelings of guilt for not achieving set goals [17, 82]. The underchallenged profile seems to be isolated, and this feeling could be increased because of their coping style, based mainly on cognitive avoidance [20], which could lead to their strong lack of engagement, and feelings of guilt as a result of the ambivalence they feel at work and their desire for change. The worn-out profile seems to be suffering the consequences of over-identification from their negative job-related thoughts and experiences, which could affect their ability to empathize with others in a functional manner, facilitating the appearance of fatigue and inefficacy, and perpetuating the symptoms of the syndrome through feelings of guilt associated with not accomplishing their responsibilities [83, 84]. However, we have seen that the explained variance of the models was relatively low, so it is necessary to continue investigating other possible sources of variability.

## Conclusions

The typological definition of the burnout syndrome through the BCSQ-36, showed a good structure and appropriate internal consistence in Spanish primary healthcare professionals. Each burnout profile showed different features and relationships with other health-related variables, which reinforces the need for treating them differently. The negative self-compassion dimensions might play a relevant role in the development of the burnout subtypes in this population. They should be considered as specific types of vulnerability when designing interventions and treatments tailored to the different characteristics of the burnout sub-types. Therefore, the overall benefit of this model is the possibility of treating each case of burnout according to its specific sources of distress. A direction for future research might be to try to provide healthcare professionals with self-compassion resources by means of mindfulness and compassion practices, for instance, for the frenetic subtype, through consulting their inner critical voice; for the underchallenged, through labelling emotions against emptiness; and in the case of the worn-out, through focusing on discomfort with equanimity. In all cases, mindfulness and self-compassion practices should focus on alleviating the specific sources of distress, guilt and other negative feelings, and they may be ideally delivered by brief online procedures which are adapted to their working hours and which provide convenience and cost-effectiveness.

## Supporting Information

S1 Fig “Burnout Clinical Subtype Questionnaire” (BCSQ-36). (DOC)

S2 Fig Fix indices for the CFA of the BCSQ-36 and models of reliability. (DOCX)
