# Correction: Burnout Subtypes and Absence of Self-Compassion in Primary Healthcare Professionals: A Cross-Sectional Study

**DOI:** 10.1371/journal.pone.0231370

**Published:** 2020-04-01

**Authors:** Jesus Montero-Marin, Fernando Zubiaga, Maria Cereceda, Marcelo Marcos Piva Demarzo, Patricia Trenc, Javier Garcia-Campayo

The following information is missing from the Funding statement: This study has been funded by Instituto de Salud Carlos III through the project “RD12/0005/0006” (Co-funded by European Regional Development Fund “Una manera de hacer Europa”).
