# Supplementary material for: Burnout Subtypes and Absence of Self-Compassion in Primary Healthcare Professionals: A Cross-Sectional Study
Source: PLoS One. 2016 Jun 16;11(6):e0157499. doi: 10.1371/journal.pone.0157499 (PMC4911164; doi:10.1371/journal.pone.0157499)
Supplement: S2 Fig — (DOCX) [file pone.0157499.s002.docx]

**S2 Fig.: Fix indices for the CFA of the BCSQ-36 and models of reliability**

| **Scales/Factors** | | **R** | **CMIN** | | **NPAR** | **GFI** | **AGFI** | **RSMR** | **NFI** | **RFI** |
| --- | --- | --- | --- | --- | --- | --- | --- | --- | --- | --- |
|  | |  |  | |  |  |  |  |  |  |
| ***Frenetic*** | |  | 216.85 | | 27 | 0.99 | 0.98 | 0.04 | 0.98 | 0.98 |
| Congeneric |  | 0.84 | 1,747.53 | 24 | | 0.91 | 0.88 | 0.12 | 0.85 | 0.81 |
| Tau-equivalent |  | 0.84 | 2,522.71 | 13 | | 0.87 | 0.85 | 0.14 | 0.78 | 0.77 |
| Parallel |  | 0.84 | 2,819.29 | 2 | | 0.86 | 0.86 | 0.14 | 0.75 | 0.78 |
|  | |  |  | |  |  |  |  |  |  |
| Ambition | |  |  | |  |  |  |  |  |  |
| Congeneric |  | 0.85 | 6.35 | 8 | | 0.99 | 0.99 | 0.02 | 0.99 | 0.99 |
| Tau-equivalent |  | 0.85 | 12,49 | 5 | | 0.99 | 0.99 | 0.03 | 0.99 | 0.99 |
| Parallel |  | 0.85 | 19,14 | 2 | | 0.99 | 0.99 | 0.03 | 0.99 | 0.99 |
|  | |  |  | |  |  |  |  |  |  |
| Overload | |  |  | |  |  |  |  |  |  |
| Congeneric |  | 0.81 | 0.26 | 8 | | 0.99 | 0.99 | 0.01 | 0.99 | 0.99 |
| Tau-equivalent |  | 0.81 | 46.81 | 5 | | 0.99 | 0.99 | 0.05 | 0.99 | 0.98 |
| Parallel |  | 0.81 | 62.00 | 2 | | 0.99 | 0.99 | 0.05 | 0.98 | 0.99 |
|  | |  |  | |  |  |  |  |  |  |
| Involvement | |  |  | |  |  |  |  |  |  |
| Congeneric |  | 0.76 | 7.88 | 8 | | 0.99 | 0.99 | 0.03 | 0.99 | 0.98 |
| Tau-equivalent |  | 0.75 | 28.36 | 5 | | 0.99 | 0.98 | 0.06 | 0.97 | 0.97 |
| Parallel |  | 0.75 | 66.57 | 2 | | 0.98 | 0.97 | 0.04 | 0.95 | 0.95 |
|  |  |  |  |  | |  |  |  |  |  |
|  | |  |  | |  |  |  |  |  |  |
| ***Underchallenged*** | |  | 104.78 | | 27 | 0.99 | 0.99 | 0.03 | 0.99 | 0.99 |
| Congeneric |  | 0.94 | 190.54 | 24 | | 0.99 | 0.99 | 0.05 | 0.99 | 0.99 |
| Tau-equivalent |  | 0.94 | 974.24 | 13 | | 0.97 | 0.97 | 0.10 | 0.96 | 0.96 |
| Parallel |  | 0.94 | 1,328.05 | 2 | | 0.96 | 0.96 | 0.07 | 0.95 | 0.96 |
|  | |  |  | |  |  |  |  |  |  |
| Indifference | |  |  | |  |  |  |  |  |  |
| Congeneric |  | 0.87 | 0.09 | 8 | | 0.99 | 0.99 | 0.01 | 0.99 | 0.99 |
| Tau-equivalent |  | 0.87 | 23.35 | 5 | | 0.99 | 0.99 | 0.05 | 0.99 | 0.98 |
| Parallel |  | 0.87 | 45.26 | 2 | | 0.99 | 0.98 | 0.04 | 0.98 | 0.98 |
|  | |  |  | |  |  |  |  |  |  |
| L. Development | |  |  | |  |  |  |  |  |  |
| Congeneric |  | 0.86 | 7.17 | 8 | | 0.99 | 0.99 | 0.02 | 0.99 | 0.99 |
| Tau-equivalent |  | 0.85 | 67.31 | 5 | | 0.99 | 0.98 | 0.06 | 0.98 | 0.98 |
| Parallel |  | 0.85 | 151.28 | 2 | | 0.98 | 0.97 | 0.05 | 0.96 | 0.97 |
|  | |  |  | |  |  |  |  |  |  |
| Boredom | |  |  | |  |  |  |  |  |  |
| Congeneric |  | 0.87 | 5.26 | 8 | | 0.99 | 0.99 | 0.02 | 0.99 | 0.99 |
| Tau-equivalent |  | 0.86 | 32.43 | 5 | | 0.99 | 0.99 | 0.04 | 0.99 | 0.99 |
| Parallel |  | 0.86 | 108.32 | 2 | | 0.98 | 0.98 | 0.03 | 0.97 | 0.98 |
|  |  |  |  |  | |  |  |  |  |  |
|  | |  |  | |  |  |  |  |  |  |
| ***Worn-out*** | |  | 400.62 | | 27 | 0.99 | 0.98 | 0.06 | 0.98 | 0.97 |
| Congeneric |  | 0.88 | 1,131.81 | 24 | | 0.96 | 0.95 | 0.12 | 0.94 | 0.93 |
| Tau-equivalent |  | 0.87 | 3,470.63 | 13 | | 0.89 | 0.86 | 0.15 | 0.82 | 0.81 |
| Parallel |  | 0.87 | 4,637.56 | 2 | | 0.85 | 0.85 | 0.12 | 0.76 | 0.79 |
| L. Acknowledgement | |  |  | |  |  |  |  |  |  |
| Congeneric |  | 0.78 | 31.16 | 8 | | 0.99 | 0.98 | 0.03 | 0.99 | 0.97 |
| Tau-equivalent |  | 0.77 | 339.77 | 5 | | 0.96 | 0.93 | 0.11 | 0.91 | 0.89 |
| Parallel |  | 0.77 | 464.27 | 2 | | 0.95 | 0.94 | 0.09 | 0.87 | 0.90 |
| Neglect | |  |  | |  |  |  |  |  |  |
| Congeneric |  | 0.86 | 1.82 | 8 | | 0.99 | 0.99 | 0.02 | 0.99 | 0.99 |
| Tau-equivalent |  | 0.86 | 5.37 | 5 | | 0.99 | 0.99 | 0.03 | 0.99 | 0.99 |
| Parallel |  | 0.86 | 24.26 | 2 | | 0.99 | 0.99 | 0.03 | 0.98 | 0.99 |
| L. Control | |  |  | |  |  |  |  |  |  |
| Congeneric |  | 0.81 | 3.25 | 8 | | 0.99 | 0.99 | 0.01 | 0.99 | 0.99 |
| Tau-equivalent | | 0.81 | 30.99 | | 5 | 0.99 | 0.99 | 0.03 | 0.99 | 0.99 |
| Parallel |  | 0.81 | 100.93 | 2 | | 0.99 | 0.99 | 0.02 | 0.97 | 0.98 |

R: Reliability; CMIN: mínimum value of the discrepancy; NPAR: number of parameters being estimated; GFI: Goodness of Fit Index; RSMR: Root Mean Square of the Standardized Residuals; AGFI: Adjusted Goodness of Fit Index; NFI: Normed Fit Index; RFI: Relative Fit Index.
